# Potential Role of Insulin Growth-Factor-Binding Protein 2 as Therapeutic Target for Obesity-Related Insulin Resistance

**DOI:** 10.3390/ijms22031133

**Published:** 2021-01-24

**Authors:** Hatim Boughanem, Elena M. Yubero-Serrano, José López-Miranda, Francisco J. Tinahones, Manuel Macias-Gonzalez

**Affiliations:** 1 Department of Endocrinology and Nutrition, Institute of Biomedical Research Institute in Malaga (IBIMA), Virgen de la Victoria University Hospital, 29010 Málaga, Spain; h.b.boughanem@gmail.com; 2 Lipids and Atherosclerosis Unit, Maimonides Institute for Biomedical Research in Cordoba (IMIBIC), Reina Sofia University Hospital, University of Córdoba, 14004 Córdoba, Spain; helese35@hotmail.com (E.M.Y.-S.); md1lomij@uco.es (J.L.-M.); 3 CIBEROBN (CIBER in Physiopathology of Obesity and Nutrition), Instituto de Salud Carlos III, 28029 Madrid, Spain

**Keywords:** IGFBP2, obesity, insulin resistance, lifestyle modification, epigenetic

## Abstract

Evidence from observational and in vitro studies suggests that insulin growth-factor-binding protein type 2 (IGFBP2) is a promising protein in non-communicable diseases, such as obesity, insulin resistance, metabolic syndrome, or type 2 diabetes. Accordingly, great efforts have been carried out to explore the role of IGFBP2 in obesity state and insulin-related diseases, which it is typically found decreased. However, the physiological pathways have not been explored yet, and the relevance of IGFBP2 as an important pathway integrator of metabolic disorders is still unknown. Here, we review and discuss the molecular structure of IGFBP2 as the first element of regulating the expression of *IGFBP2*. We highlight an update of the association between low serum IGFBP2 and an increased risk of obesity, type 2 diabetes, metabolic syndrome, and low insulin sensitivity. We hypothesize mechanisms of IGFBP2 on the development of obesity and insulin resistance in an insulin-independent manner, which meant that could be evaluated as a therapeutic target. Finally, we cover the most interesting lifestyle modifications that regulate IGFBP2, since lifestyle factors (diet and/or physical activity) are associated with important variations in serum IGFBP2.

## 1. Introduction

Insulin-like growth factors (IGFs) are growth peptides, that are implicated in mammalian development, growth and cell proliferation and differentiation [1]. IGFs are usually bound to IGF-binding proteins (IGFBPs), and mediate their actions by regulating their bioavailability [2]. There are six well characterized high-affinity IGFBP members, designated IGFBP1 through 6. The IGFBP family members share similar structure and molecular organization, which is suggestive of similar mechanism of action, whereas they have different modes of regulation and distinct expression patterns [3]. In this wise, IGFBP2 is a key member that participates in different physiological and metabolic processes.

The physiological role of IGFBP2 on metabolic disorders are controversial and less defined, although there is growing evidence for a solid association [4]. Therefore, understanding metabolic regulation of IGFBP2 and its influence on metabolic diseases could provide new insights that can be applied as therapeutic targets. In this review, we highlight the genetic and molecular structure of IGFBP2 to better understand its metabolic function, genetic regulation, as well as physiological role. We provide an update of the association between low serum IGFBP2 and an increased risk of obesity, metabolic syndrome, type 2 diabetes, as well as insulin resistance. We hypothesize mechanisms by which IGFBP2 could be implicated in the development of obesity and insulin resistance as well. We finally review the main risk factors that are associated with low serum levels of IGFBP2, since these factors may be considered for the prevention strategies and treatment of obesity-related insulin resistance. 

## 2. Building the Molecular Structure of IGFBP2

**Figure 1:**
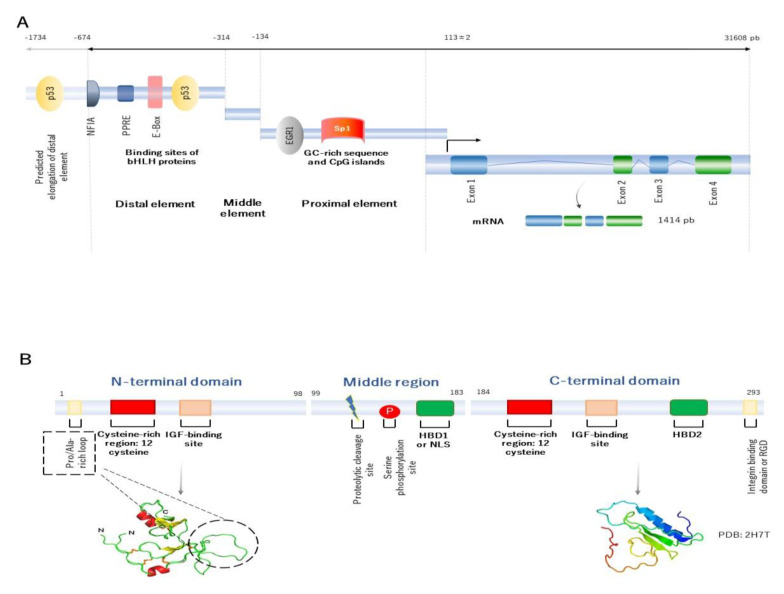
Human representative of *IGFBP2* genetic and molecular structure. (**A**) Human representative of genetic structure of *IGFBP2*. There are three regulatory elements, ranging from distal, middle to proximal elements. The structure gene contains four exons and three introns. The product is a mature mRNA of 1414 pb. (**B**) Human representative structure of IGFBP2 protein domain for the mature protein of 289 amino acids residues. There are three principal domains. The N-terminal domain, the middle domain or the middle region and the C-terminal domain. The N- (extracted from Galea C. et al. [9]) and C-terminal (PDB: 2H7T for the C-domain of IGFBP2) domain are presented. Both N- and C-terminal domains contain IGF binding sites, which suggests that these domains are highly conserved. However, the middle region has a proteolytic cleavage and phosphorylation sites, which indicates that this domain may be implicated in the regulation of bioavailability of IGF1 and stability of the protein structure.

The human *IGFBP2* gene is located in chromosome 2q35 and has four exons and three introns [5,6]. The combination of exons and introns provides up to five splice variants. However, only the first transcript variant encodes for the functional isoform, that it is usually found to be involved in its main physiological role [7]. The *IGFBP2* gene expression is modulated by the interaction of a number of transcription factors at the regulatory elements in the promoter region. The *IGFBP2* promoter is located between the position -674 upstream up to the transcription start site (TSS), localized into 113 ± 2 [5,8]. In 2006, Sato and collaborators, by studying dedifferentiated lung cancer cells, suggested that the structure of the human *IGFBP2* promoter can be organized into three elements, represented as the distal (localized between −674 and −314, which apparently acts as an enhancer element), middle (extends from −314 to −134, and seems to be an inhibitor/silencer), and proximal regulatory element (extends from −134 to the transcription start site, and apparently acts as a regulator of transcriptional activity). It is worth mentioning that the proximal regulatory element lacks TATA or CAAT boxes and contains high-GC sequences, meaning that this element may participate in epigenetic regulation (Figure 1A) [8]. 

In human, a wide number of transcription factors can bind to the *IGFBP2* promoter and activate its transcription activity in vitro. In 2006, Sato et al. and Grimberg et al. found by analyzing lung cancer cells, that the *IGFBP2* promoter has putative binding sites for early growth response protein 1 (EGR1) and transformation-related protein 53 (p53), that are crucial for its transactivation [8,10]. In addition, in small-cell lung cancer cells, Yazawa et al. (2009) demonstrated that neurogenic differentiation (NEUROD)—a helix-loop-helix (HLH) transcription factor—binds to the enhancer box (E-box) sequence, located at the distal element and regulates its activation [11]. Further, Mireuta and colleagues (2010) argued that the proximal regulatory element is a potential target for specificity protein 1 (Sp1) [12]. In 2017, Lee and collaborators showed in MCF-7 epithelial breast tumor cells, that nuclear factor IA (NFIA) binds to the E-box sequence, to potentially enhance *IGFBP2* transcription as well [13]. Recently, our group, by studying nuclear extracts from visceral adipose tissue, demonstrated that peroxisome-proliferator activated receptor γ2 (PPARγ2) (through PPARγ2-retinoid X receptor α (RXRα) heterodimer) physically interacts with the *IGFBP2* promoter through PPAR responsive element (PPRE) domain [14]. 

Accordingly, comparative studies in animal models reveal a large number of metabolic transcription factors that can bind to the *igfbp2* promoter, such as multiple endocrine neoplasia type 1 (MEN1), Sp1, PPARα, CCAAT-enhancer-binding protein α (C/EBPα), or hypoxia-inducible factor 1 (HIF1) and nuclear factor kappa B (NFκB) [15]. Therefore, these transcription factors could predict the physiological role of IGFBP2, at least at transcriptional and transductional levels. The fact that the *IGFBP2* has PPAR, NEUROD, and NFκB binding sites places it as an important mediator of metabolic and inflammatory processes. Conversely, the notion of the interaction with transcription factors, such as p53, sp1, EGR1, and MEN1, provides an idea of its role in cell cycle, cell proliferation, and growth. 

Overall, the *IGFBP2* gene encodes for a final mature IGFBP2 protein, that contains 289 amino acid residues of 31.4 kDa of molecular weight. Its structure has three principal domains: the N-terminal, middle region, and the C-terminal domain. The N- and C-terminal domains are conserved regions between the IGFBP family members, whereas the middle region shows high variability. This region, therefore, contributes for a singular identity and specific functions (Figure 1B).

The N-terminal domain of IGFBP2 protein, encoded by the exon 1, contains 98 amino acid residues and shares approximately 58% of sequence between the IGFBP family members. This domain is characterized by containing a highly conserved cysteine-rich region, which allows to form up to six disulphide bonds. The N-domain contains an additional 16-residue Pro/Ala-rich, situated between Pro21 and Arg36 residues, that is not found in the other members. This motif forms a solvent-exposed loop on the three-dimensional structure of the protein, and may comprise a potential -SH3 domain interaction site for binding to other proteins [9]. Within this domain, another local motif (GCGCCxxC) is found, which may be crucial in the interaction of IGFBP2 with IGFs [9]. The N-terminal domain has an IGFs-binding domain, that is involved in high-affinity binding to IGFs [9].

The middle region (called L-domain) is encoded by the exon 2 and links the N- to the C-terminal domains. This domain extends approximately for 85 amino acids residues, and its sequence appears to be unique and singular among the IGFBP members. It is susceptible to proteolytic cleavage and inactivation of IGFBP2 protein, since specific proteases separate the N- and C-domains, and decrease the affinity of IGFBP2 by IGFs. The middle region has various predicted phosphorylated sites [16], and it is often phosphorylated in serine at the position 106 [17]. The purpose of phosphorylation is still unknown, but may contribute to the resistance to proteolysis. Within the L-domain, there is another subdomain called heparin-binding domain 1 (HBD1), located between 179 and 184. The principal function of the HBD1 domain is to bind to extracellular matrix components (as glycosaminoglycans, integrins, etc.). The HBD1 domain can act as a ligand, by binding to the tyrosine phosphatase β (RPTPβ) receptor [18], and mimic the nuclear localization signal (NLS) to translocate into the nucleus as well [19]. Therefore, this motif is required for specific recognition and interaction binding.

Finally, the C-terminal region is encoded by the exon 3 and 4 and contains 107 amino acid residues. Interestingly, the sequence of this domain shares high similarity with the thyroglobulin-type-I domain [20]. This domain is conserved between IGFBP members and characterized by highly conserved six cysteine residues [21]. The main function of C-domain is involved in high-affinity binding to IGFs, which contains a specific region to bind to IGFs. Within this domain, an RGD (Arg-Gly-Asp) sequence, located on 265 and 267 residues, can bind to integrins [22], and activate many physiological and cell cycle processes, such as cell invasion and proliferation, by inducing β-catenin and further affecting Wnt signaling [23]. The C-terminal domain contains an HBD motif as well, called HBD2. The interaction of HBD2 to proteoglycans leads to regulate many of cell cycle processes as cell adhesion, proliferation, and migration [24].

Classically, the molecular structure of IGFBP2 is designed to bind to IGFs, but also can act as a ligand, through different domains located within the protein. This fact places IGFBP2 as a versatile molecule that could participate in a wide range of metabolic and cell cycle processes. Nonetheless, despite the aforementioned findings, many of the molecular pathways of IGFBP2 remain unknown. Investigating the molecular pathways of IGFBP2 could help to better understand the role that IGFBP2 plays in metabolic diseases.

## 3. Mechanism of Action of IGFBP2 and its Physiological Role

**Figure 2:**
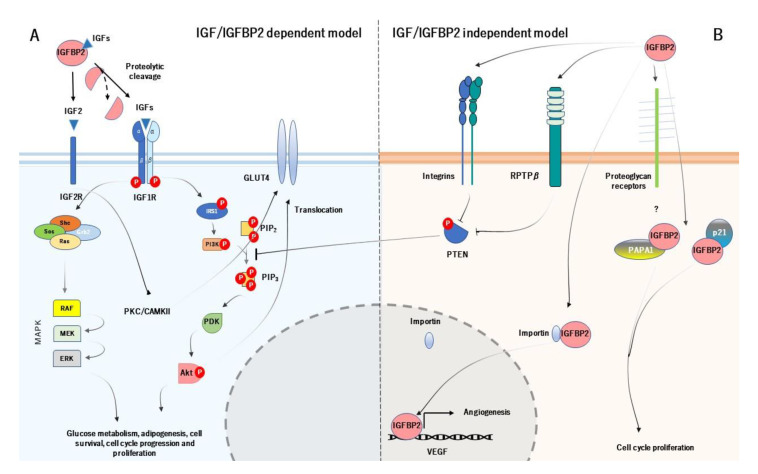
The cellular action of IGFBP2 is mediated by two principal models. The IGF-dependent model (**A**) modulates the activity and bioavailability of IGFs, which prevents the binding of IGFs to their receptor. The IGF-independent model (**B**) constitutes the physical interaction between IGFBP2 and specific receptors at the cell surface and extracellular matrix, which acts as a ligand. The main function of both models is regulating cell cycle, insulin, and glucose metabolism and angiogenesis.

IGFBP2 acts systematically following two main action models, known as IGF-dependent model (IGFBP2 binds and regulates the bioavailability of IGFs) and IGF-independent model (IGFBP2 acts as a ligand by binding to receptors at the cell surface and to extracellular matrix) (Figure 2) [25]. Likewise, the principal function of IGFBP2 starts with its binding to IGFs (they form a binary complex) and modulates their actions at systemic levels. The colocalization of IGF type 1 or 2 with IGFBP2 has frequently been questioned, suggesting a possible leaning for each one. Interestingly, IGFBP2 has more pronounced affinity for IGF2, up to 10-20-fold greater than IGF1, which may be its primary ligand [26]. The binding of IGFBP2 to IGF1 seems to locally modulate IGF1 signaling, under various conditions [27]. However, as discussed below, the biological meaning of the IGF2/IGFBP2 complex is to increase its affinity by the extracellular matrix [28].

The IGF-dependent model is responsible for the regulation of the phosphatidylinositol 3-kinase (PI3K)/ alpha serine/threonine-protein kinase (Akt) signalling pathway. The activation of PI3K/Akt pathway starts with the specific proteolysis and release of IGFBP2 from IGF1 or IGF2 and the binding of IGFs to IGF1 receptor (IGF1R) [29]. The complex IGFs/IGF1R leads to conformational changes in the IGF1R, which auto-phosphorylates their subunits β. The auto-phosphorylation of the IGF1R causes the activation of PI3K, which further induces the phosphorylation and activation of Akt. The active Akt has a wide number of target genes related with cell cycle and glucose metabolism, such as mechanistic target of rapamycin complex 1 (mTORC1), BCL2 associated agonist of cell death (BAD) and translocation of glucose transporter 4 (GLUT4), and also inhibits a large number of genes, as tuberous sclerosis 1 (TSC1) or glycogen synthase kinase 3 β (GSK3β) [30]. On the other hand, the phosphorylated IGF1R can also induce the recruitment of SHC adaptor protein (Shc), growth factor receptor-bound protein 2 (Grb2), Son of Sevenless (SOS), and rat sarcoma (Ras), which leads to stimulate the mitogen activated protein kinase (MAPK) signalling pathway. The MAPK pathway is responsible for growth processes, such as development, cell proliferation, differentiation, and migration, as well as inhibition of apoptosis [31] (Figure 2A). As for the IGF2R, it has been thought that it does not have apparent intracellular signalling activity and it is believed to act as a scavenger receptor for IGF2 [32]. However, a study carried out by Chu et al. (2008), showed that IGF2R in H9c2 cardiomyoblast cell cultures, activates the protein kinase C (PKC)/calcium/calmodulin-dependent protein kinase II (CaMKII) signalling pathway, which is dependent on the IGF2R [33]. This may result in GLUT4 translocation to the plasma membrane, characterizing an insulin-independent pathway [34].

As for the IGF-independent model, IGFBP2 acts as a potential ligand that binds to different targets. In particular, IGFBP2 binds to integrins through RGD domain, and stimulates cell migration and proliferation, adhesion, as well as cell differentiation [35]. The activation of IGFBP2/integrin complex increases the phosphorylation and inactivation of phosphatase and tensin homolog (PTEN), which leads to the stimulation of Akt signaling [36] (it is worth noting that despite the IGFBP2/integrin inactivates PTEN, there are also other mechanisms that regulate PTEN in this context). Moreover, IGFBP2 binds to extracellular matrix, which this interaction is mediated by HBD. Its biological function is to modulate the bioavailability of IGFs in the extracellular space and also acts as an active and local reservoir of IGFs to regulate cell adhesion, migration, proliferation, and differentiation [24,37]. The HBD domain is used to bind to RPTPβ receptor, being a receptor with tyrosine phosphatase activity. The IGFBP2/RPTPβ leads to its dimerization and further inactivates its phosphatase function. The inactivation of RPTPβ, cooperatively with IGF1 triggers to increase the phosphorylation of PTEN and further stimulate Akt [38]. In addition, the binding of IGF2 to IGFBP2 greatly increases the affinity for O-sulfated glycosaminoglycans, heparin, and heparan sulfate. Accordingly, Lund et al. (2014) demonstrated in vitro that the HBD1 and HBD2 contribute differentially to glycosaminoglycans binding and increase the affinity of both free IGFBP2 and the IGF2/IGFBP2 protein complex for heparin [28]. 

Interestingly, IGFBP2 can be located within the cells as well. A study pointed out that IGFBP2 intracellularly interacts with cyclin-dependent kinase inhibitor 1 (p21), to modulate cell proliferation in vivo in mouse lung epithelial cell line [39]. IGFBP2 also interacts with Pim-1-associated protein-1 (PAPA1) in the human prostate cancer cell line (LNCaP). The binding of IGFBP2 to PAPA1 seems to have a role in growth-promoting effect. The inhibition of PAPA1 enhances cell growth, suggesting that the proliferative effect of IGFBP2 may be regulated by its intracellular interaction with PAPA1 [40]. But IGFBP2 may act at intranuclear levels. A study demonstrated that IGFBP2 can be located into the nucleus in several common cancer cells. This is possible because of the HBD1 motif (HBD1 can mimic the NLS domain). Within the nucleus, IGFBP2 can act as a transcription modulator, which contributes to the activation of the expression of vascular endothelial growth factor (VEGF), an essential gene for angiogenesis [19] (Figure 2B).

As already noted, IGFBP2 modulates the IGF signaling, binds to the extracellular matrix, and interacts with the cell surface receptor to modulate the PI3K/Akt signaling pathway. Given that IGFBP2 regulates the bioavailability of IGFs, it is worth noting that IGFBP2 is a key factor in cell proliferation and growth. The IGFs/IGFBP2 is closely related with glucose/insulin metabolism (through PTEN/Akt signaling) and cell cycle (trough MAPK pathway) as well, which could explain the dual role of IGFBP2 to act in such processes. Thus, the metabolic role of IGFBP2 could place it as a potential regulator in metabolic disorders, either by IGFs/IGFBP2-dependent and independent model.

## 4. IGFBP2 and Obesity-Related Insulin Resistance

IGFBP2 is considered a major regulator of IGFs bioavailability in metabolic signaling pathway. Recent studies show solid association between mRNA and serum IGFBP2 and metabolic disorders, including obesity, metabolic syndrome, insulin resistance, and type 2 diabetes, as detailed below. However, although the mechanisms by which IGFBP2 participates in the development of obesity-related diseases are still controversial, there is more and more evidence for a solid association between serum IGFBP2 and these metabolic disorders.

### 4.1. The Suggested Role of IGFBP2 in the Development of Obesity

The majority of studies reached a consensus idea that serum IGFBP2 is decreased in obesity state, which is observed in different state of obesity and closely related to other anthropometric variables. In 1997, Nam et al. investigated the effect of obesity on the serum levels of IGFBP2 in 88 males. This study concluded that IGFBP2 concentrations were suppressed in 43 obese subjects than in 45 normal controls [41]. Indeed, this effect is manifested at early stages. Both Ballerini et al. (2004) and Yau et al. (2018) analyzed serum IGFBP2 in children with obesity. These studies found that serum IGFBP2 was decreased in obese children [42], and was negatively associated with body mass index (BMI), anthropometric variables, markers of metabolic dysfunction, blood pressure, and insulin sensitivity [43]. Likewise, a prospective study with mean follow-up of 6.2 years, conducted in 2009 by Hu et al. in 625 participants, confirmed the inverse correlation between serum IGFBP2, obesity and adiposity, since higher serum IGFBP2 are associated with lower adiposity and decreased glucose tolerance [44]. Another study conducted in 38 prepubertal obese children, by Claudio and colleagues (2010) showed that *IGFBP2* expression in subcutaneous adipose tissue biopsies was associated with fat mass percentage and adiposity, insulin sensitivity, free IGF1 and leptin, suggesting a close relationship between IGFBP2 and adiposity, independently from the level of insulin sensitivity [45]. 

Furthermore, it is noteworthy to note that IGFBP2 has a protective role in obesity. This evidence was confirmed by a recent study, conducted by Ceccarini et al. (2019) in 51 patients with obesity. These patients had lower IGFBP2 levels compared with 41 lean matched controls. After gastric bypass, serum IGFBP2 increased at 3 days and maintained normal concentrations before the occurrence of relevant changes in body weight, and remained stable up to 18 months after surgery. IGFBP2/leptin ratio also increased early after surgery and return normal after one year [46]. Consistently, a recent study conducted by Al-Regaiey et al. (2020) confirmed the previous findings, showing that serum IGFBP2 increases in 33 obese patients after bariatric surgery and further weight loss [47]. Thus, these studies strongly manifest that IGFBP2 are decreased in obesity state, and suggest that IGFBP2 might be important in the pathogenesis of obesity. These findings point out to protective effects, which further in vitro experiments may clarify the physiological mechanism for this protection.

It is worth pointing out that IGFBP2 could prevent obesity through inhibition of adipogenesis according to dependent and independent action models. An in vitro study demonstrated that IGFBP2 prevents adipogenesis, through inhibition of 3T3-L1 cells differentiation and modulation IGF1 activity [48]. It is known that IGF1 mediates adipocyte growth and differentiation, which is known to be increased when preadipocytes differentiate into mature adipocytes. In addition, HBD domains also mediate the inhibitory effect on preadipocyte differentiation [49]. Both HBD1 and HBD2 peptides inhibit preadipocyte differentiation, although the HBD2 peptide is more effective in in vitro and in vivo IGFBP2^−/−^ mice. The administration of HBD2 to IGFBP2^−/−^ mice reduced gain in total fat mass and visceral fat accumulation. The HBD2 peptide also increased serum leptin, suggesting that HBD2 domain of IGFBP2 is the primary region that accounts for its ability to inhibit adipogenesis [49]. Indeed, IGFBP2 seems to be more important in visceral adipose tissue than in subcutaneous adipose tissue. Accordingly, a study reported that *IGFBP2* DNA methylation is increased in visceral adipose tissue than in subcutaneous adipose tissue in 24 obese subjects, suggesting an epigenetic regulation of *IGFBP2* in abdominal obesity [50].

IGFBP2 not only modulates adipogenesis process, but also acts as an adipokine. Hedbacker et al. (2010) showed that leptin-deficient patients (*n* = 3) are associated with decreased serum IGFBP2 in comparison with age- and weight-matched controls. However, after six months with leptin treatment, these patients recovered normal circulating IGFBP2 levels. In addition, the treatment of ob/ob mice with leptin induces the expression of *IGFBP2*, suggesting a close crosstalk between IGFBP2 and leptin [51]. Leptin is an essential hormone that can regulate adipogenesis, and directly increases the expression of *IGFBP2* in human skeletal muscle cells. This effect is carried out by both signal transducer and activator of transcription-3 (STAT3) and PI3K signaling pathways, to enhance insulin signaling. However, when *IGFBP2* is silenced, it leads to the decrease of leptin and insulin-stimulated protein kinase B (PKB) phosphorylation as well as glucose uptake. In in vivo experiments, central leptin infusion upregulates the expression of *IGFBP2* in skeletal muscle, liver, subcutaneous and visceral fat in sheep infused with intracerebroventricular leptin. This animal model also improves glucose tolerance and serum insulin levels after glucose load. Therefore, leptin regulates IGFBP2, which probably has an impact on peripheral insulin sensitivity and glucose metabolism [52]. Overall, Neumann et al. (2014) showed that IGFBP2 acts as a mediator of leptin on obesity and insulin sensitivity, by restoring metabolic variables and weight/BMI in ob/ob mice in a similar mechanism of action of leptin. However, IGFBP2 alone is not sufficient to mimic the physiological effect of leptin [53]. 

The action of IGFBP2 in obesity appears to act by both dependent (by modulating IGFs) and independent models (through HBD domains) by directly inhibiting adipogenesis and differentiation of preadipocytes at locally visceral adipose tissue. This action helps to reduce fat mass and weight gain. There is a cross-talk between leptin and IGFBP2. The aforementioned studies suggest that IGFBP2 may be permissive for obesity development, since its role in the pathogenesis of obesity is for long-term. Then, IGFBP2 acts rather as a potential collaborator/mediator of leptin, than a principal actor. 

### 4.2. The Mechanisms of IGFBP2 in Insulin Sensitivity

Overall, the abovementioned studies demonstrate that the robust effects of IGFBP2 on obesity are fully reproducible across humans, different animal, and cell line models. The notion that IGF/IGFBP2 system are involved in the insulin system pathway, opens the possibility to think that IGFBP2 might be related to insulin sensitivity. However, many unsolved issues still hinder the role of IGFBP2 in insulin sensitivity and insulin resistance, and the suggested the association with type 2 diabetes, which we discuss below.

Clemmons et al. (1991) reported that serum IGFBP2 did not show significant fluctuation under physiological/metabolic and nutritional conditions, such as post-prandial state or administration of glucose. However, a long time of 9 fasting days—as a result of insulin deficiency—caused an increased serum IGFBP2 level in seven obese subjects, suggesting that insulin may have a physiological role in regulating serum levels of IGFBP2 [54]. Arafat and colleagues (2009) demonstrated that insulin increases IGFBP2 in 24 healthy subjects and 19 subjects with impaired glucose tolerance. In addition, subjects with impaired glucose tolerance showed more pronounced insulin resistance and lower serum IGFBP2 levels in comparison with healthy control. IGFBP2 was an independent predictor of insulin sensitivity, which suggests that IGFBP2 plays a central role in the insulin/IGFs system crosstalk and it is closely linked to insulin resistance [55]. In this line, our group (2017) found that both mRNA and IGFBP2 protein in visceral adipose tissue were decreased in 13 morbid obese patients with high insulin resistance when compared with in 12 morbid obese patients with low insulin resistance [56]. 

Interestingly, in 2018, Yau et al. investigated the relationship between serum IGFBP2 and insulin sensitivity in 194 children with obesity. This study found a positive association between insulin sensitivity index-homeostasis model assessment and serum IGFBP2 [43]. In this line, our group (2019) also determined, by studying nuclear extract from visceral adipose tissue, that the activation of *IGFBP2* promoter by PPARγ2-RXRα heterodimer was decreased in 11 high insulin resistance patients with morbid obesity, when compared with in 12 low insulin resistance patients [14]. Recently, Van den Beld and colleagues (2019) conducted a 20-year longitudinal study in the Baltimore Longitudinal Study of Aging cohort in 539 participants, where it showed that the serum IGFBP2 was positively correlated with insulin sensitivity and inversely with BMI, both at baseline and follow-up [57].

As noted, these studies demonstrate that is a robust association between IGFBP2 and insulin and glucose tolerance, which indicates that IGFBP2 may regulate insulin sensitivity and glucose metabolism through distinct molecular pathways and/or target organs. Accordingly, in 2012, Li et al. demonstrated in vitro that *IGFBP2* mRNA and protein concentration in serum-deprived 3T3-L1 adipocytes were increased by acute insulin treatment. Further treatments with PI3K or mTOR inhibitors blunted the effects of insulin, suggesting that insulin upregulates *IGFBP2* expression through a PI3K/mTOR/C/EBPα pathway in white adipocytes [58]. Additionally, Yau and collaborators (2014) reported that leptin directly increases *IGFBP2* mRNA and protein in human skeletal muscle cells through PI3K signaling, in parallel with enhanced insulin signaling, and silencing *IGFBP2* lowered leptin- and insulin-stimulated PKB phosphorylation and glucose uptake [52]. In addition, in lipoma cells PTEN-deficient, Wilhelm et al. (2015) demonstrated that IGFBP2 production was not influenced, either by inhibition of mTORC1 and MAPK. However, the inhibition of PI3K decreases *IGFBP2* expression and secretion [59]. Accordingly, IGFBP2 significantly increases the activation through phosphorylation of PI3K, Akt, AMP-activated protein kinase (AMPK), and PKC in 3T3-L1 adipocytes and induced GLUT4 translocation, improving glucose intake. Moreover, Assefa and colleagues (2017) demonstrated that IGFBP2 stimulates glucose uptake in 3T3-L1 adipocytes through activation of PI3K/Akt, AMPK/TBC1 Domain 1 (TBC1D1), and PI3K/PKC/GLUT4 signaling pathways. IGFBP2 actions were independent of its binding to IGF1 and is possibly not mediated through the insulin or IGF1 receptor [60]. Therefore, all these studies point out that IGFBP2 improves insulin sensitivity through increased glucose uptake and that synergistic activation of PI3K/Akt and AMPK mediates the modulatory effect of IGFBP2 in both dependent and independent actions. It is worth noting that the interaction of IGFBP2 with IGF2 modulates the binding with IGF2 to their receptor. Overall, the activation of IGF2R activates PKC/CAMKII signaling pathway, and further leads to the translocation of GLUT4 and the improvement of glucose uptake [33,34].

### 4.3. The Association of IGFBP2 and Type 2 Diabetes

Regarding the role of IGFBP2 in type 2 diabetes, Rajpathak et al. (2012) found in 742 type 2 diabetes patients and 742 matched control subjects, that IGFBP2 concentration was decreased in type 2 diabetes patients, in comparison with control participants, and higher serum IGFBP2 was associated with lower risk of type 2 diabetes [61]. IGFBP2 is not only associated with type 2 diabetes, but also with gestational diabetes. A cross-sectional study, conducted by Lappas and colleagues (2016), found in 98 patients with gestational diabetes mellitus, after adjusting with age and BMI, that serum IGFBP2 was significantly associated with the development of type 2 diabetes [62], indicating that low postpartum IGFBP2 levels are a significant risk factor for the development of type 2 diabetes in women with a previous history of gestational diabetes. In 2017, a study revealed possible mechanism by which higher serum IGFBP2 decreases the risk of developing type 2 diabetes. Further, several longitudinal studies, meta-analyses and systematic reviews confirmed in different cohorts that IGFBP2 is found decreased in type 2 diabetes and supposes a factor risk for development of type 2 diabetes [63,64]. Recently, Wittenbecher et al. (2019) showed that IGFBP2 was associated with lower risk of type 2 diabetes in 755 cases versus 2778 controls, and DNA methylation of the *IGFBP2* gene was also associated with higher type 2 diabetes risk, suggesting an epigenetic alteration of the *IGFBP2* gene in the type 2 diabetes context [65]. Finally, a recent proteome-wide study, conducted by Noordam et al. (2020) found that IGFBP2 protein was found lower among 175 type 2 diabetes patients compared with 164 controls [66]. 

As for the *IGFBP2* polymorphisms, several studies demonstrated that single nucleotide polymorphisms (SNP) in the *IGFBP2* gene are strongly associated with type 2 diabetes. For instance, Horikawa et al. (2008) found that SNP rs1470579 *IGFBP2* was associated with significant type 2 diabetes risk in 1900 Japanese patients, with odds ratio (OR) of 1.18 (Confidence interval (CI): 1.07–1.31) [67]. In the same year, another genome-wide study conducted by Cauchi and colleagues, found an increased risk of rs1470579 allele risk and type 2 diabetes in 6890 French population (OR = 1.17; CI: 1.07–1.27) [68]. In 2010, Huang et al. also found a significant association between rs1470579 and an increased risk of type 2 diabetes, and these polymorphisms may affect the therapeutic efficacy of repaglinide in 350 Chinese patients [69]. In contrast, Duesing and collaborators were unable to replicate the confirm rs1470579 susceptibility variant with type 2 diabetes in a case-control cohort comprising 3093 French Caucasian subjects, probably due to the nature of study design [70]. Therefore, there is increasing evidence of the association of polymorphisms of *IGFBP2* and type 2 diabetes. However, further genetic and functional studies are needed to clarify the implication of these polymorphisms on the type 2 diabetes contribution. 

Therefore, the aforementioned findings suggest that IGFBP2 shares a promising association with insulin resistance and type 2 diabetes. However, its functional role is needed to be clarified. The role of IGFBP2 in type 2 diabetes may be related with regulation of IGFs, glucose uptake, and PI3K/Akt pathway, which indicates that IGFBP2 may have a specific role in the pathway of insulin resistance, either by dependent- or independent-IGF signaling pathway.

### 4.4. IGFBP2 Is Associated with Metabolic Syndrome

Metabolic syndrome is a cluster of well-defined conditions, that include increased blood pressure, high blood glucose, obesity, characterized by increased abdominal fat, and increased circulating lipid levels, such as cholesterol and triglycerides. Metabolic syndrome patients have typically increased risk of type 2 diabetes.

IGFBP2, as a potential risk factor of insulin sensitivity, is related with metabolic syndrome. Heald et al. (2006) reported in 163 type 2 diabetes patients that 125 of those patients with metabolic syndrome patients presented lower levels of IGFBP2 than those in 38 non-metabolic syndrome participants [4]. This study also showed that low serum IGFBP2 was associated with elevated fasting glucose, triglycerides, and low-density lipoprotein (LDL)-cholesterol and positively correlated with insulin sensitivity, which could be a possible biomarker for metabolic syndrome. The notion that supports the implication of IGFBP2 with metabolic syndrome is that low IGFBP2 levels are associated with a deleterious lipid profile. Accordingly, Carter and colleagues (2014) reported that low serum IGFBP2 levels were associated with impaired insulin sensitivity, increased fat mass and higher plasma triglycerides, by studying 379 Caucasian men. In addition, serum IGFBP2 levels were significantly and independently associated with very LDL-triglycerides levels, which concludes in a metabolic alterations, being an early dyslipidemia but also for main cluster for metabolic syndrome [71]. But IGFBP2 is also associated with a deleterious blood pressure profile. Olszanecka and collaborators (2017) reported that 152 hypertensive women had significantly lower serum IGFBP2 levels than 40 non-hypertensive women and was negatively correlated with 24-h systolic blood pressure. Indeed, this study showed a negative correlation with a number of metabolic syndrome components [72]. Moreover, a recent study, conducted by Pouriamehr et al. (2019) confirmed that 110 metabolic syndrome patients have a lower level of IGFBP2 compared with subjects in the control group [73].

As already mentioned, the role of IGFBP2 on metabolic syndrome needs to be studied in depth. The association of IGFBP2 with metabolic syndrome variables may explain in part its potential role in developing metabolic disease. The role of IGFBP2 in glucose intake, insulin sensitivity, including insulin resistance, lipid profile, and obesity may contribute to metabolic syndrome. Anyway, higher concentration of IGFBP2 at baseline was associated with a lower risk of incident metabolic syndrome [74], and vice versa. 

### 4.5. Proposed Mechanisms of IGFBP2 in Obesity-Related Insulin Resistance

**Figure 3:**
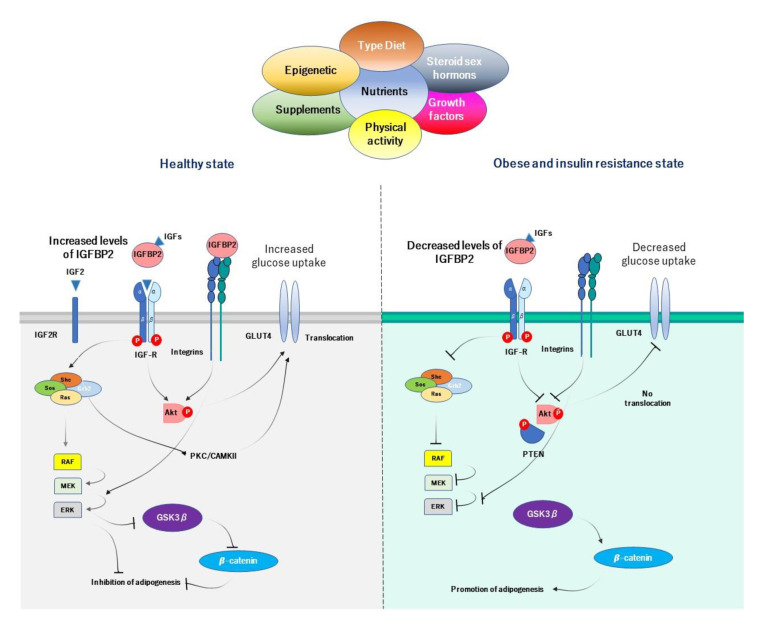
Proposed mechanism of action of IGFBP2 in obesity and insulin sensitivity. Dietary components and lifestyle modifications could affect circulating levels and mRNA expression of *IGFBP2*. The combination of these factors could provide a functional role of IGFBP2 on several metabolic disorders, and assign different strategies of prevention. In health state, higher levels of IGFBP2 act in both dependent and independent models to inhibit adipogenesis and activate the translocation of GLUT4, and improve the insulin sensitivity. In obese and insulin resistant state, lower IGFBP2 levels are the results of multiple factors, such as diet, sedentary, and low component and supplement from diet. When IGFBP2 is decreased, the adipogenesis process is activated and the translocation of GLUT4 is reduced, which results in increased body fat and poor insulin sensitivity.

Our proposed mechanism by which IGFBP2 exerts preventive properties in obesity, begins with the ability that IGFBP2 has to prevent and inhibit adipogenesis at local visceral adipose tissue. Adequate serum levels of IGFBP2 inhibit differentiation of preadipocytes, and further hyperplasia and hypertrophy of visceral adipose tissue in both dependent and independent actions, which results in preventing obesity state (Figure 3). The action of IGFBP2 could occur at IGF-dependent manner, in which IGFBP2 modulates the action of IGFs in preadipocytes, by inhibiting adipogenesis. It has been demonstrated that IGF1 stimulates proliferation of 3T3-L1 preadipocytes through activation of MAPK, which is mediated through the Src family of nonreceptor tyrosine kinases [75]. Likewise, the action of IGFBP2 through IGF-independent pathway is related to its interaction with integrin. A study reported that knockdown of integrin reduced the proliferation of human adipose tissue stem cells, and promoted adipogenic differentiation. This action is carried out by the RGD domain and its interaction with integrins, suggesting a negative impact of RDG-motif signaling on adipogenic differentiation of human adipose tissue stem cells via integrin [76,77]. Integrin activates rapidly accelerated fibrosarcoma (RAF) and then extracellular signal-regulated kinase (ERK). ERK inhibits GSK3β, which in turn inhibits β-catenin and adipogenesis [76]. The HBD2 domain also may reduce gain in total fat mass and visceral fat accumulation and inhibits adipogenesis [49].

In obese state, a combination of risk factors leads to reduce serum IGFBP2 levels. Unhealthy way of life, including overeating, hypercaloric diet, high protein diet, and low consumption of carbohydrates and unsaturated fat, as well as low physical activity, has been linked to decreased IGFBP2 circulating levels. Low IGFBP2 levels do not regulate adequately the local action of IGFs at adipose tissue levels, resulting in increasing rate of adipogenesis by IGFs. In addition, a low activity of integrin at preadipocytes decreases the activity of RAF and ERK, inhibits GSK3β, and activates β-catenin, then results in the progression of the adipogenesis process. HBD2 does not exert its correct function by inhibiting adipogenesis at correct rate as well. 

As regarding to insulin sensitivity and insulin resistance, the proposed mechanism by which IGFBP2 improves insulin sensitivity, is through IGFs/IGFBP2-dependent and independent model. IGFs has the ability to activate the translocation of GLUT4 through the activation of Akt, and when IGFBP2 binds to integrins, this interaction results in the phosphorylation of PTEN and inactivation of their functions. PTEN acts as a negative regulator of insulin signaling in skeletal muscle and adipocytes. The inhibition of PTEN increases the activity of PI3K, which results in the translocation of GLUT4 from cytoplasm to surface cell. Thus, IGFBP2 participates in the regulation of glucose metabolism and insulin sensitivity through PI3K/Akt activity by an independent-manner of IGFs (Figure 3). The improvement of insulin sensitivity through IGFBP2 is dose-IGFBP2 dependent, in which adequate levels of IGFBP2 mark the action of both insulin and IGFs on glucose uptake into the cells. In addition, the activation of IGF2R by the modulation of IGF2/IGFBP2 leads to the translocation of GLUT4 and improves glucose uptake [33,34].

## 5. Diverse Strategies to Modify Serum IGFBP2 in a Prevention Context

It has been demonstrated that many human tissues express *IGFBP2* gene. *IGFBP2* expression can be detected in brain, blood, mature adipocytes, and tumoral cells. In particular, IGFBP2 is highly expressed in the pancreas and liver, but liver and adipocytes are the most interesting tissues because of their role in the metabolism. Thus, the expression pattern of *IGFBP2* is regulated under a cell-specific pathway, which its expression control is subject to a fine regulation between diet, lifestyle, and epigenetic interactions. All together, they regulate the expression of *IGFBP2*. 

### 5.1. Diet and Lifestyle Modifications

The main function of IGFBP2, therefore, might be related with metabolic effect such as insulin, glucose, and lipid metabolism. Table 1 resumes the main nutrients that modulate the concentration of IGFBP2 in serum. Pucilowska and colleagues showed in 1993, that IGFBP2 value before refeeding was twice in 22 children, and decreased after a high protein diet [78], although these children were infected with shigellosis. In 1995, Fouque et al. evaluated a 3-month controlled study in 12 adult chronic renal failure patients with low protein diet. After a 4- to 6-week equilibrium period, serum IGFBP2 was significantly higher in chronic renal failure patients than normal adults [79]. In addition, in 2007, Vrieling and collaborators conducted a clinical trial in 40 men and 31 postmenopausal women with lycopene and green tea supplementation and found these supplements significantly increased serum IGFBP2 concentrations in both men and women than after placebo [80]. In 2009, Crowe et al. reported in a cross-sectional study of 4731 men and women, that the intake of carbohydrate, in particular, the intake of starch by not free sugar, and monounsaturated fat content were associated with increased IGFBP2 in plasma, whereas total and dairy animal protein were related to decreased IGFBP2 [81], which was further validated in 2012 in another cross-sectional study conducted by Young et al. [82]. Additionally, a study conducted in 2016 reported that the fruits and vegetables consumption in 265 mid-childhood and 261 adolescence was related to higher IGFBP2 in adulthood [83]. Nonetheless, other nutrients also modulate the bioavailability of IGFBP2. A cross-sectional proteomic study (n = 16) conducted in 2018 showed that circulating vitamin D levels, upper to 50 nmol/L, are correlated with increased plasma levels of IGFBP2 in men with obesity [84].

These aforementioned findings suggest that IGFBP2 seems to be particularly sensitive to diet. Therefore, a personalized diet strategy could be considered beneficial for health, related to IGFBP2 levels. Accordingly, there is growing evidence that personalized diet could modify serum IGFBP2. In 2002, a cross-sectional study of 292 British women conducted in a plant-based (vegan) diet showed that the mean concentrations of serum IGFBP2 were 20–40% higher in vegan women compared to meat-eaters and vegetarians [93]. In 2005, 34 obese subjects were subjected to 8 weeks with very low-calorie diet (VLCD), followed by 12 weeks with a weight-stabilizing diet. After 20 weeks, serum IGFBP2 was increased in comparison with baseline [94]. A few years later, in 2012, Touskova et al. confirmed these results, showing that after VLCD, 13 obese women with type 2 diabetes showed increased *IGFBP2* expression in subcutaneous adipose tissue [95]. Recently, in 2019, Carter and colleagues conducted a longitudinal one-year lifestyle modification program consisting in personalized healthy eating and physical activity counseling, combined to elicit a daily 500 kcal deficit. The intervention triggered a 43% increase of IGFBP2 levels in 99 participants, and subjects with the most substantial increases in IGFBP2 also experienced the most important metabolic improvements [96].

In summary, results from the previous studies of dietary factors and the concentrations of IGFBP2 show a number of potentially important associations. The key findings were that higher intakes of protein and low carbohydrate and monounsaturated fats were related to decreased concentrations of serum IGFBP2. These associations may be important to understand the etiology of IGFBP2 in metabolic disorders associated with diet. 

### 5.2. The Effect of Physical Activity on Serum IGFBP2

Physical activity is a key modifier of IGFBP2, since it is considered a potential modulator of circulating IGFBP2. The endurance exercise training shows to have an effect on the mRNA expression of *IGFBP2* in skeletal muscle. Kopple et al. demonstrated in 2006, that *IGFBP2* expression was increased at the end of exercise training in 10 sedentary maintenance hemodialysis patients who underwent exercise [97]. In 2008, Berg et al. evaluated the effect of ultra-endurance exercise on serum IGFBP2. Results showed an increasing serum IGFBP2 levels in only 7 endurance athlete women after 24 hours [91], suggesting a possible relationship with female hormone such as estradiol, since previous studies reported that estrogen is a negative regulator of IGFBP2 in plasma [98]. 

Otherwise, endurance training also was able to increase IGFBP2, by Gregory and colleagues (2013) [92]. Indeed, an interaction between diet and exercise are noted in a previous study conducted by Foster et al. (2012) [99]. Eight college-aged males completed three high-intensity interval training protocols followed by three post-exercise nutritional protocols with placebo, carbohydrate only, and essential amino acid/carbohydrate. Significant differences were noted in IGFBP2 only in the placebo group, in which IGFBP2 was significantly increased. Likewise, Nindl et al. (2016) evaluated differential responses of the IGF system in resistance exercise-based program, during an acute resistance exercise protocol in 33 volunteers. This study found that after 4 and 8 weeks of training, serum IGFBP2 was significantly increased in comparison with baseline value [100]. Accordingly, a recent study confirmed the previous results. Four weeks of high-intensity exercise training resulted in a statistically significant change in the plasma level of IGFBP2 and further eight months of intensive exercise also resulted in a significant increase of IGFBP2 [101].

As already noted, diverse exercise strategies are commented to modulate the levels of serum IGFBP2. These strategies may be taken account in the management of many chronic noncommunicable diseases, such as type 2 diabetes and obesity. A strategic plan and modification of lifestyle, which contain balanced diet (high carbohydrates and monounsaturated fat, moderate-protein intake) and physical activity (both endurance and resistance exercise) could be essential in the design study of evaluating serum IGFBP2, and also as preventive strategies in non-communicable diseases.

### 5.3. Epigenetic Regulation also Influences IGFBP2

The epigenetic landscape of *IGFBP2* is still premature and need to be assessed with caution. However, there is evidence supporting the idea that epigenetic changes at the promoter of *IGFBP2* cause strong modification in the *IGFBP2* expression and further IGFBP2 bioavailability. In 2005, Chiba et al. showed aberrant methylation of *IGFBP2* in hepatoma cancer, linking *IGFBP2* as a tumor suppressor gene [102]. In this line, Sato and colleagues (2006) illustrated that *IGFBP2* promoter has CpG islands located at the proximal regulatory elements, which is frequently methylated [8]. There is a CpG-rich region, which it is proposed as a genetic regulator. The transcription factor TFIID binds to CG-rich sequences in the *IGFBP2* promoter, and initiates RNA transcription. A hypermethylated status of this regions may influence the ability of these transcription factors to bind in, and further regulate the initiation of RNA transcription. Indeed, Zheng et al. (2011) found *IGBP2* hypermethylation profiles specific for glioma subtypes and it was associated with low *IGFBP2* mRNA expression [103]. Additionally, another study conducted by Ahrens et al. (2013) evaluated the DNA methylation analysis in 45 nonalcoholic fatty liver (NASH) patients, and found that IGFBP2 locus was hypermethylated in NASH, suggesting a potential role in metabolic disorders at the epigenetic levels [104]. Moreover, a study conducted by Nawathe et al. (2016) investigated the relationship between DNA methylation of components of the IGF axis in the placenta and disorders in fetal growth. This study found that the CpG methylation of the *IGFBP2* promoter was lower in the placenta from small gestational age neonates as compared to appropriately grown neonates, but was unchanged in the placenta from large gestational age neonates [105]. Interestingly, a recent study reported that silenced *IGFBP2* acts as a tumor suppressor gene in epithelial bladder cancer cells. Silencing *IGFBP2* by siRNA promotes cell proliferation and invasion. This study showed that *IGFBP2* was epigenetically silenced via DNA methylation. The exposition of cancer cells to methylation inhibitor (5-Aza-2′-Deoxycytidine (AZA)), produced re-expression of IGFBP2, and increased in abundance of IGFBP2, indicating that *IGFBP2* could be epigenetically silenced [106]. In the same years, Zhang et al. showed that *IGFBP2* DNA methylation levels in visceral adipose tissue were increased in obese subjects, and increased when compared to the *IGFBP2* DNA methylation in subcutaneous adipose tissue, irrespective of obesity [50], suggesting a specific-depot epigenetic alteration in the IGFBP2 regulation.

At histone modification levels, different molecules use diverse strategies to increase IGFBP2 levels. Biernacka et al. (2013) reported that glucose increased IGFBP2 via increasing the acetylation status of histones H3 and H4 associated with the *IGFBP2* gene promoter [107]. In 2015, Pickard et al. demonstrated that using 3-dimensional organotypic cultures, that repression of *IGFBP2* is mediated by histone deacetylation at the *IGFBP2* promoter and was reversed by treatment with histone deacetylase (HDAC) inhibitors [108]. Likewise, Phillip and collaborators (2016) further reported that deficient-*IGFBP2* glioma cell treatment with IGFBP2 resulted in a rapid increase of lysine-specific demethylase 1 (LSD1) and methylated histones that have been identified as direct targets of LSD1 (H3K9 and H3K27). The process has been shown to be reversible, suggesting a possible role of *IGFBP2* at epigenetic regulation and specifically affected epigenetic factors. [109]. Indeed, the inhibition of LSD1 increases the methylation levels of H3K4 at the promoter site of *IGFBP2*, indicating that *IGFBP2* is regulated under histone modification levels [110]. However, the inactivation of histone deacetylase sirtuin 6 (SIRT6) acts as histone deacetylation at *IGFBP2* promoter and inhibits *IGFBP2* expression. Haploinsufficiency of SIRT6, but not complete loss of SIRT6 promotes *IGFBP2* expression via increased chromatin accessibility, by H3K56 acetylation at the *IGFBP2* locus. Overall, modification at histone levels in the promotor of *IGFBP2* increases cell development and cell survival [111].

Therefore, the promoter of *IGFBP2* is highly regulated by epigenetic modifications, indicating that *IGFBP2* is sensible to epigenetic reprogramming. It is worth mentioning that epigenetic mechanism on IGFBP2 was not linked with a determined phenotype. However, nutrients were widely associated with epigenetic reprogramming. Therefore, the interaction of epigenetic landscape of IGFBP2 with nutritional compounds, diet, and lifestyle modifications may be a topic of interest.

## 6. Preclinical Pharmacology Studies of IGFBP2

IGFBP2 has potent beneficial therapeutic action in obesity and insulin resistance. To explore the effect of IGFBP2 on the development of diet-induced obesity and its metabolic consequences, Weathcroft et al. (2007) evaluated the effect of high-fat/high calorie diet for 32 weeks in IGFBP2 transgenic mice. Weight gain on the high-fat diet was attenuated in transgenic mice compared with wild-type mice. Wild-type mice markedly increased their fat depots, whereas IGFBP2 transgenic mice remained lean. In addition, blood glucose after a glucose tolerance test was significantly lower in IGFBP2 than wild-type mice on both types of diet, suggesting that IGFBP2 protects against obesity and insulin resistance [48]. Likewise, Hedbacker et al. (2010) evaluated the effect of IGFBP2 in obesity and insulin sensitivity. IGFBP2 was injected into streptozotocin-induced insulin-deficient mice. At day 5 after IGFBP2 injections, control mice had increased fasting glucose compared to the IGFBP2-treated group. The glucose tolerance test was also markedly improved at 45 min. Otherwise, the ob/ob expressed-IGFBP2 mice showed decreased weight and food intake, decrease fasting glucose and insulin and glucose tolerance [51].

Despite its multiple metabolic benefits, human IGFBP2 in its native form may be not enough suitable for clinical use owing to its pharmacokinetic profiles. In addition, native IGFBP2 is susceptible to proteolytic cleavage in the middle region and further inactivation. As suggested, HBD2 peptide is more effective in in vivo and IGFBP2^−/−^ mice. The administration of HBD2 to IGFBP2^−/−^ mice reduced gain in total fat mass and visceral fat accumulation. The HBD2 peptide also increased serum leptin, suggesting that HBD2 domain of IGFBP2 is the primary region that accounts for its ability to inhibit adipogenesis [49]. Therefore, various biopharmaceutical engineering approaches may be adopted to develop IGFBP2 analogues and mimetics with improved physiological properties in obesity and insulin sensitivity, pharmacokinetic profiles and potency that are suitable for future clinical development.

## 7. Concluding Remarks and Future Perspectives

The major conclusions that can be drawn from this review are that IGFBP2 is sensitive to relevant changes in our lifestyle. There are evidences that serum levels vary with large number of diet component and physical activity. Thus, restoring IGFBP2 levels by a lifestyle modification program or by new therapeutic strategies could be interesting, in which increased levels of IGFBP2 are associated with improvements of metabolic variables in obesity and insulin sensitivity. Indeed, IGFBP2 could be also interesting to use as an early marker and monitoring for several metabolic diseases, such as obesity, metabolic syndrome, type 2 diabetes, or insulin resistance. In this line, serum IGFBP2 concentrations could allow for scientists and physicians to monitor the improvement of lifestyle modification and treatment intervention. However, further studies are needed to determine a consensus modification lifestyle program. The current perspective is that IGFBP2 is decreased in metabolic disorders, in which several risk factors lead to decreased IGFBP2 levels, under different molecular targets, including epigenetic, transcriptional, and protein levels. Future works are needed to establish new strategies to use IGFBP2 in anti-obesity and related diseases treatment. In addition, we also need to understand many of IGFBP2 pathways that are implicated, since more knowledges about this issue could help us to find new strategies pathway in metabolic diseases. Our opinions about the role of IGFBP2 in obesity-related insulin resistance research, is that we may consider a cohort that has controlling lifestyle habits, diet and supplements, and new therapeutic options. It is also important to consider biochemical parameters and value the effect of antidiabetic on IGFBP2 concentrations to understand better the role of IGFBP2 in non-communicable diseases.

## Figures and Tables

**Table 1:** Dietary and lifestyle components that alter the circulating levels of IGFBP2.

Groups	Effectors	Effects of IGFBP2	Physiological Context	Refs
Nutrients	Vitamin D	**↑**	Men with obesity with high vitamin D had increased IGFBP2 levels than lower vitamin D subjects	[84]
	Protein	**↓**	High protein diet was associated with decreased serum IGFBP2 levels	[81]
	Calcium	**↓**	High calcium diet was associated with decreased serum IGFBP2 levels	[81]
	Carbohydrates	**↑**	High carbohydrates diet was associated with increased serum IGFBP2 levels	[81]
	Monounsaturated fat	**↑**	High monounsaturated diet was associated with increased serum IGFBP2 circulating levels	[81]
	Lycopene and green tea	**↑**	A randomized, placebo-controlled, double-blinded crossover study showed that lycopene and green tea supplements were associated with increased serum IGFBP2	[80]
	Fruits and flavonoid intake	**↑**	High fruits and flavonoid intake was associated with increased serum IGFBP2 circulating	[83]
Growth factors	Insulin	**↑**	Insulin increased IGFBP2 in cultured embryonic kidney cell line	[85]
	IGF1, IGF2 and IGF analogues	**↑**	IGF1, IGF2 and IGF analogues increase IGFBP2 levels in human subjects and different cell models	[6,85,86,87,88,89,90]
	Leptin	**↑**	Leptin stimulates expression of *IGFBP2* and increases protein levels in human skeletal muscle cells	[52]
Physical activity	Endurance and resistance exercises	**↑**	Physical activity was associated with increased serum IGFBP2	[91], [92]

## Data Availability

Data sharing not applicable.

## Abbreviations

p53	transformation-related protein 53
NFIA	nuclear factor IA
PPRE	peroxisome-proliferator activated receptor responsive element
E-Box	enhancer box
bHLH	Basic helix-loop-helix
EGFR1	epidermal growth factor receptor 1
Sp1	specificity protein 1
IGF1	insulin growth factor type 1
HBD1	heparin binding domain 1
NLS	nuclear localization sequence;
HBD2	heparin binding domain
RGD	tripeptide Arg-Gly-Asp
PDB	Protein data bank
IGFBP2	insulin growth factor binding protein 2.
IGF2	Insulin growth factor 2;
IGF2R	Insulin growth factor receptor type 2
IGF1R	Insulin growth factor receptor type 1
Shc	SHC Adaptor Protein
SOS	Son of Sevenless
Ras	rat sarcoma
Grb2	growth factor receptor-bound protein 2
IRS1	Insulin receptor substrate 1
PI3K	phosphatidylinositol 3-kinase
PIP2	Phosphatidylinositol 4,5-bisphosphate
PIP3	Phosphatidylinositol 3,4,5-trisphosphate
GLU4	glucose receptor type 4
MAPK	mitogen activated protein kinase
RAF	Rapidly Accelerated Fibrosarcoma
MEK	Mitogen-activated protein kinase kinase
ERK	extracellular signal-regulated kinase
PKC/CAMKII	protein kinase C/calcium/calmodulin dependent protein kinase II
PDK	Pyruvate dehydrogenase kinase
Akt	alpha serine/threonine-protein kinase
RPTPβ	tyrosine phosphatase β
PAPA1	Pim-1-associated protein-1
P21	cyclin-dependent kinase inhibitor 1
VEGF	vascular endothelial growth factor
GSK3β	glycogen synthase kinase 3 β
